# Pfs230 yields higher malaria transmission–blocking vaccine activity than Pfs25 in humans but not mice

**DOI:** 10.1172/JCI146221

**Published:** 2021-04-01

**Authors:** Sara A. Healy, Charles Anderson, Bruce J. Swihart, Agnes Mwakingwe, Erin E. Gabriel, Hope Decederfelt, Charlotte V. Hobbs, Kelly M. Rausch, Daming Zhu, Olga Muratova, Raul Herrera, Puthupparampil V. Scaria, Nicholas J. MacDonald, Lynn E. Lambert, Irfan Zaidi, Camila H. Coelho, Jonathan P. Renn, Yimin Wu, David L. Narum, Patrick E. Duffy

**Affiliations:** 1 Laboratory of Malaria Immunology and Vaccinology, and; 2 Biostatistics Research Branch, National Institute of Allergy and Infectious Diseases, National Institutes of Health, Bethesda, Maryland, USA.; 3 Department of Medical Epidemiology and Biostatistics, Karolinska Institute, Stockholm, Sweden.; 4 Pharmacy Department, Clinical Center, National Institutes of Health, Bethesda, Maryland, USA.

**Keywords:** Infectious disease, Vaccines, Malaria

## Abstract

**BACKGROUND:**

Vaccines that block human-to-mosquito *Plasmodium* transmission are needed for malaria eradication, and clinical trials have targeted zygote antigen Pfs25 for decades. We reported that a Pfs25 protein-protein conjugate vaccine formulated in alum adjuvant induced serum functional activity in both US and Malian adults. However, antibody levels declined rapidly, and transmission-reducing activity required 4 vaccine doses. Functional immunogenicity and durability must be improved before advancing transmission-blocking vaccines further in clinical development. We hypothesized that the prefertilization protein Pfs230 alone or in combination with Pfs25 would improve functional activity.

**METHODS:**

Transmission-blocking vaccine candidates based on gamete antigen Pfs230 or Pfs25 were conjugated with Exoprotein A, formulated in Alhydrogel, and administered to mice, rhesus macaques, and humans. Antibody levels were measured by ELISA and transmission-reducing activity was assessed by the standard membrane feeding assay.

**RESULTS:**

Pfs25-EPA/Alhydrogel and Pfs230D1-EPA/Alhydrogel induced similar serum functional activity in mice, but Pfs230D1-EPA induced significantly greater activity in rhesus monkeys that was enhanced by complement. In US adults, 2 vaccine doses induced complement-dependent activity in 4 of 5 Pfs230D1-EPA/Alhydrogel recipients but no significant activity in 5 Pfs25-EPA recipients, and combination with Pfs25-EPA did not increase activity over Pfs230D1-EPA alone.

**CONCLUSION:**

The complement-dependent functional immunogenicity of Pfs230D1-EPA represents a significant improvement over Pfs25-EPA in this comparative study. The rhesus model is more predictive of the functional human immune response to Pfs230D1 than is the mouse model.

**TRIAL REGISTRATION:**

ClinicalTrials.gov NCT02334462.

**FUNDING:**

Intramural Research Program of the National Institute of Allergy and Infectious Diseases, National Institutes of Health.

## Introduction

The world has achieved substantial strides in malaria control, with roughly half of the countries endemic for malaria having eliminated the disease in the past 50 years. However, existing tools failed to achieve elimination despite comprehensive application in African settings (1, 2), and recent progress to reduce malaria cases has stalled globally and been reversed in some areas (3). Vaccines have been essential for elimination of infectious agents such as smallpox, polio, and measles by halting the onward transmission of these diseases, conferring major benefits to human health and economies (4–6). Malaria transmission-blocking vaccines (TBVs) were conceived in the 1970s as a tool to interrupt parasite transmission with antibodies that attack sexual-stage parasites in the mosquito vector (7, 8). Monoclonal antibodies to mosquito sexual-stage (gamete) parasites were used to identify candidate TBV antigens, including gamete surface proteins P230 and P48/45, first expressed by gametocytes in mammalian host blood (9), and zygote surface proteins P25 and P28, expressed only after fertilization in the mosquito host (10, 11). These antigens are multidomain cysteine-rich proteins and generally difficult to produce as properly folded recombinant protein. *Plasmodium falciparum* P25 (Pfs25) antigen was the first expressed as recombinant protein (12) and has remained the leading TBV candidate for 3 decades. In preclinical studies, Pfs25 vaccines have induced equal or greater serum activity versus other candidate antigens or antigen combinations (13, 14). TBV activity is measured in mosquito feeding assays that assess whether immune sera reduce the parasite burden (transmission-reducing activity, TRA) or the proportion of infected mosquitoes (transmission-blocking activity, TBA).

While earlier P25 candidates failed to meet safety or activity criteria to advance in the clinic (15, 16), we recently reported that a Pfs25 protein-protein conjugate vaccine formulated in alum adjuvant induced serum functional activity in both US and Malian adults (17, 18). However, few vaccinees developed TRA greater than 50% or significantly increased their TRA after 2 or 3 doses of Pfs25 vaccine. This occurred in 2 of 17 subjects after 2 doses and 2 of 15 subjects after 3 doses in US adults (17), and no significant activity was seen in vaccinees (versus comparators) after 3 doses in Malian adults (18). In each study, significant functional activity required 4 vaccine doses, and antibody levels declined rapidly, suggesting functional immunogenicity and durability must be improved before advancing TBV further in clinical development. Preclinical evidence suggested that TBV combinations might enhance vaccine activity (19), and we have proposed that Pfs25 should be assessed in combination with other antigens to improve human vaccine activity (18). Specifically, we hypothesized that the combination of Pfs230 prefertilization activity and Pfs25 postfertilization activity might exceed their individual activities.

To assess the contribution of Pfs230 to a TBV, a fragment (Ser_542_ to Gly_736_) encompassing domain 1 of Pfs230 cloned and expressed in *P*. *pastoris* (Pfs230D1, previously referred to as Pfs230D1M) as described in (20) was chemically conjugated to EPA (21), a nontoxic mutant of exoprotein A from *P*. *aeruginosa* using methods previously described for development of the Pfs25-EPA vaccine (22). Here, we compare Pfs230D1-EPA to our benchmark TBV (Pfs25-EPA) formulated in alum in 3 models (mice, nonhuman primates, and humans) and assess their activity in combination.

## Results

To confirm the benefit of conjugation of Pfs230D1, groups of CD-1 mice were immunized twice (0, 28 days) by intramuscular injection with either Pfs230D1 or Pfs230D1-EPA, both formulated in Alhydrogel, which was the clinical formulation. Antibody levels induced by Pfs230D1-EPA were approximately 100-fold greater than those induced by Pfs230D1 monomer (Supplemental Figure 1; supplemental material available online with this article; https://doi.org/10.1172/JCI146221DS1). For clinical development planning, we assessed Pfs230D1 and Pfs25 for their relative capacities to induce functional antibodies, to determine if one antigen should be prioritized over the other for clinical testing. Initial dose ranging studies with Pfs230D1-EPA were performed in mice in order to determine appropriate doses to use for future mouse experiments (Supplemental Figure 2). Subsequently, all mice were immunized with doses ranging from 0.1 μg to 3 μg per immunization 28 days apart.

**Figure 1:**
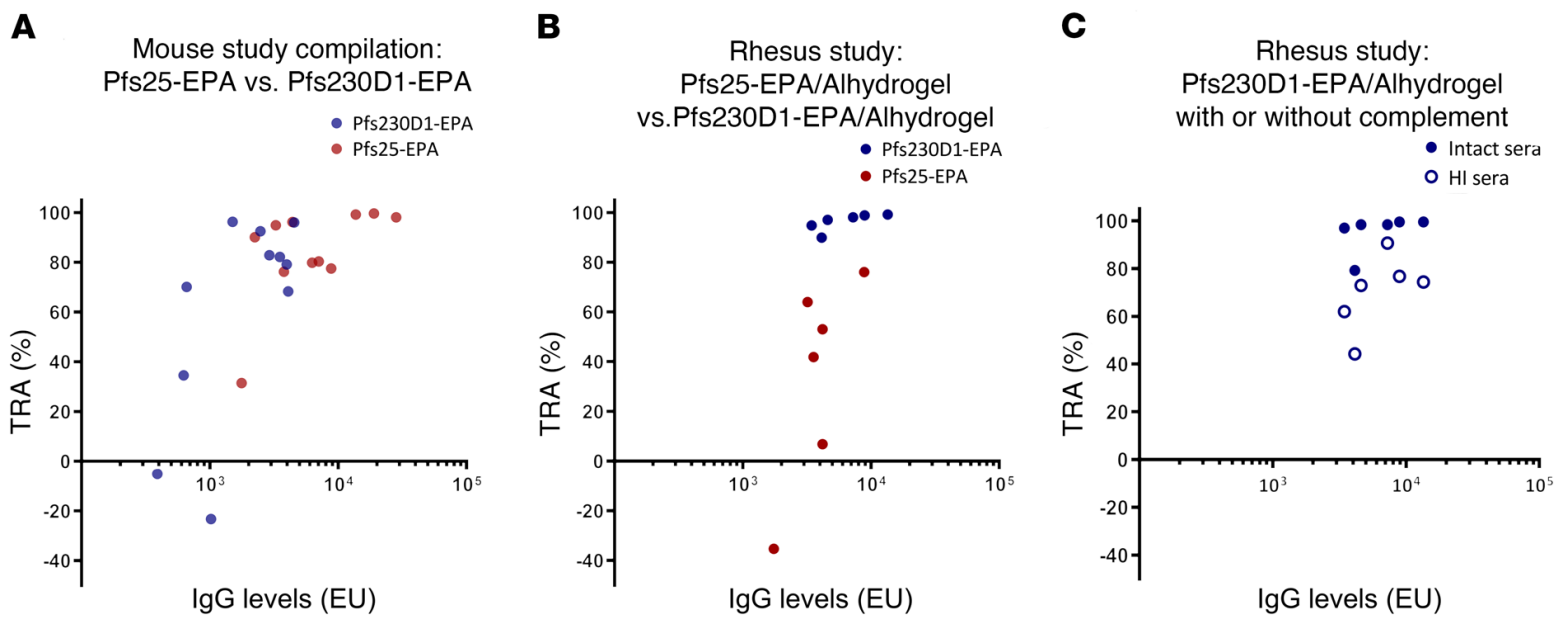
Mouse and rhesus studies comparing Pfs25-EPA/Alhydrogel with Pfs230D1-EPA/Alhydrogel. Mouse studies revealed no difference in immunogenicity or functional activity, whereas rhesus studies suggest Pfs230D1 is superior to Pfs25 and that it requires active complement. (**A**) Mouse sera samples collected after immunization with Pfs25-EPA/Alhydrogel or Pfs230D1-EPA/Alhydrogel were used in SMFA to measure antibody function. Each data point represents a pool of sera from 1 immunization group (*n =* 10/group). (**B**) Rhesus monkey sera samples collected 2 weeks after the third immunization with Pfs25-EPA/Alhydrogel or Pfs230D1-EPA/Alhydrogel were used in SMFA to measure antibody function. Each data point represents an individual animal. Percentage of TRA is relative to prebleed samples from each animal. (**C**) Sera from the Pfs230D1-EPA/Alhydrogel group were divided in 2 aliquots, allowing heat inactivation of 1 aliquot, followed by SMFA. For all panels, the antibody level shown in the *x* axis indicates the ELISA units (EU) in the mosquito feeder after dilution. Pearson’s correlation coefficient was used for the pairs of log-antibody level and TRA, stratified by vaccine antigen.

Data were collated from multiple experiments using both BALB/c and CD-1 outbred mice with head-to-head comparisons of Pfs25-EPA/Alhydrogel and Pfs230D1-EPA/Alhydrogel for functional antibody activity assessed by standard membrane feeding assay (SMFA) of *P*. *falciparum* strain NF54 gametocytes to *Anopheles stephensi* mosquitoes (Figure 1A). Parasite TRA (reduction in mean oocyst count per mosquito versus control antibody) was plotted against ELISA levels for 4 separate experiments, each containing 3 immunizing doses. There was a positive correlation for both antigens (Pfs230D1, *r* = 0.68 *P =* 0.02; Pfs25, *r* = 0.58 *P =* 0.06), with high TRA achieved at the higher ELISA levels with both antigens, but with no difference between Pfs25 and Pfs230D1.

Multiple publications have shown a dependency on active complement for anti-Pfs230 inhibition in preclinical studies (23–25). Therefore, serum samples from BALB/c mice immunized with cGMP Pfs230D1-EPA/Alhydrogel were tested by SMFA when mixed with intact or heat-inactivated human sera. Again, sera from mice immunized with Pfs230D1-EPA inhibited oocyst development in a dose-dependent manner and was not significantly different from that of Pfs25-EPA sera (Supplemental Figure 3 and Supplemental Table 1). In the absence of active human complement in a dose titration experiment, antibodies against both antigens blocked equally well.

Since mouse studies did not distinguish one antigen (Pfs25 or Pfs230D1) as superior over the other for functional activity and therefore their prioritization for clinical testing, we hypothesized a nonhuman primate model may reveal quantitative differences in activity of the 2 vaccine candidates. We conducted a vaccine study in rhesus macaques using the clinical Alhydrogel formulation and doses of Pfs230D1-EPA (40 μg Pfs230D1) and Pfs25-EPA (47 μg Pfs25) administered by intramuscular injection on a 0, 2-, 6-month schedule, similar to a typical human clinical trial regimen and schedule. Antibody responses were monitored to confirm Pfs25 and Pfs230D1 immunogenicity (Figure 1B and Supplemental Figure 4, A and C). Sera with peak antibody levels, collected 2 weeks after the third dose (day 182), were used in SMFA to assess function in the presence of intact human sera (Figure 1B, Supplemental Figure 4, B and D, and Supplemental Table 2). Anti-Pfs25 immune sera demonstrated overall modest inhibition of oocyst development, similar to previous preclinical and clinical experience. In contrast, anti-Pfs230D1 had potent inhibitory activity, with all samples reducing oocyst density by greater than 80%, and 3 of 6 achieving greater than 50% reduction in the prevalence of infected mosquitoes, referred to as TBA.

To measure the contribution of complement to the activity, serum samples from the Pfs230D1 group were tested again by SMFA in the presence or absence of complement (Figure 1C and Supplemental Table 3). Distinct from observations in mice, activity was significantly diminished in the absence of complement (*P =* 0.03, Wilcoxon matched-pairs signed rank test for difference between groups). However, even without complement, 5 of 6 monkeys had greater than 50% TRA, suggesting rhesus antibody may block a parasite function to neutralize gametes, independent of its role in complement-mediated lysis. Thus, the rhesus model indicated that Pfs230D1 was a superior vaccine target, in part due to the activity of complement.

**Figure 2:**
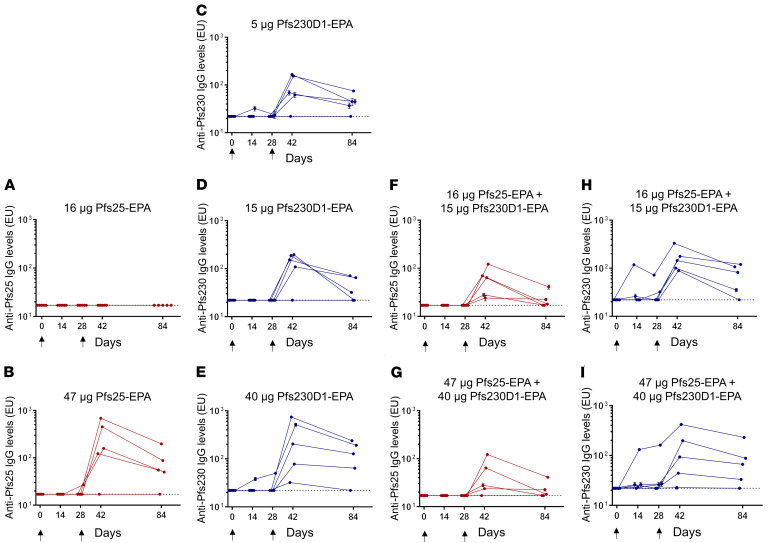
Antibody levels by ELISA in humans. Antibody levels from each vaccine arm. Vaccine dosing refers to the amount of target antigen (16 μg, 47 μg Pfs25; 5 μg, 15 μg, 40 μg Pfs230D1) contained in the total conjugate vaccine dose administered to that study arm. Vaccinations were performed on days 0 and 28 at escalating doses (*n =* 5/arm). Day 42 values were analyzed by separate Wilcoxon rank sum tests with continuity correction (**A** vs **F**, **B** vs **G**, and **A** vs **B**). Comparisons among **C**, **D**, and **E** were done using the Kruskal-Wallis rank sum test.

After establishing that Pfs230D1-EPA/Alhydrogel and Pfs25-EPA/Alhydrogel were well-tolerated, immunogenic, and induced functional activity in preclinical models, a phase 1 first-in-human clinical trial was initiated to assess safety and to compare Pfs25-EPA/Alhydrogel, Pfs230D1-EPA/Alhydrogel, or a combination of the 2, before advancing either candidate to field trials. US adults were vaccinated in a staggered manner for safety (see Supplemental Tables 8 and 9). Five subjects per arm received 2 immunizations (0, 28 days) with Pfs230D1-EPA/Alhydrogel or Pfs25-EPA/Alhydrogel, or both coadministered in separate arms. Overall, vaccinations with all doses of Pfs25-EPA/Alhydrogel (16 μg; 47 μg), Pfs230D1-EPA/Alhydrogel (5 μg; 15 μg; 40 μg), and the combination (Pfs25 + Pfs230D1: 16 μg + 15 μg; 47 μg + 40 μg) were well-tolerated with minimal local and systemic reactogenicity reported and no serious adverse events (Supplemental Table 4). Pfs25-EPA/Alhydrogel elicited anti-Pfs25 antibodies at the high dose but not at the low dose (Figure 2B compared with Figure 2A, P *=* 0.03), whereas antibody responses against Pfs230D1 were not dose-dependent (Figure 2, C–E). Combining low-dose Pfs25-EPA/Alhydrogel with low-dose Pfs230D1-EPA/Alhydrogel elicited significantly higher anti-Pfs25 responses than low-dose Pfs25-EPA/Alhydrogel alone (Figure 2F compared with Figure 2A, P *=* 0.01). This effect was not observed with high-dose vaccine, where Pfs25 showed a trend to lower antibody levels after coadministration of the 2 vaccines (Figure 2G compared with Figure 2B, P *=* 0.12), and there was no effect of vaccine combinations on anti-Pfs230 responses (Figure 2, H and I). Conjugate vaccines using the same carrier protein can have either a negative or positive interaction, and dosage may influence these interactions (26). Interestingly, anti-Pfs230 antibodies were measurable even at the lowest Pfs230D1-EPA dose of 5 μg (Figure 2C), unlike the Pfs25-EPA vaccine (18), suggestive of better intrinsic immunogenicity.

**Figure 3:**
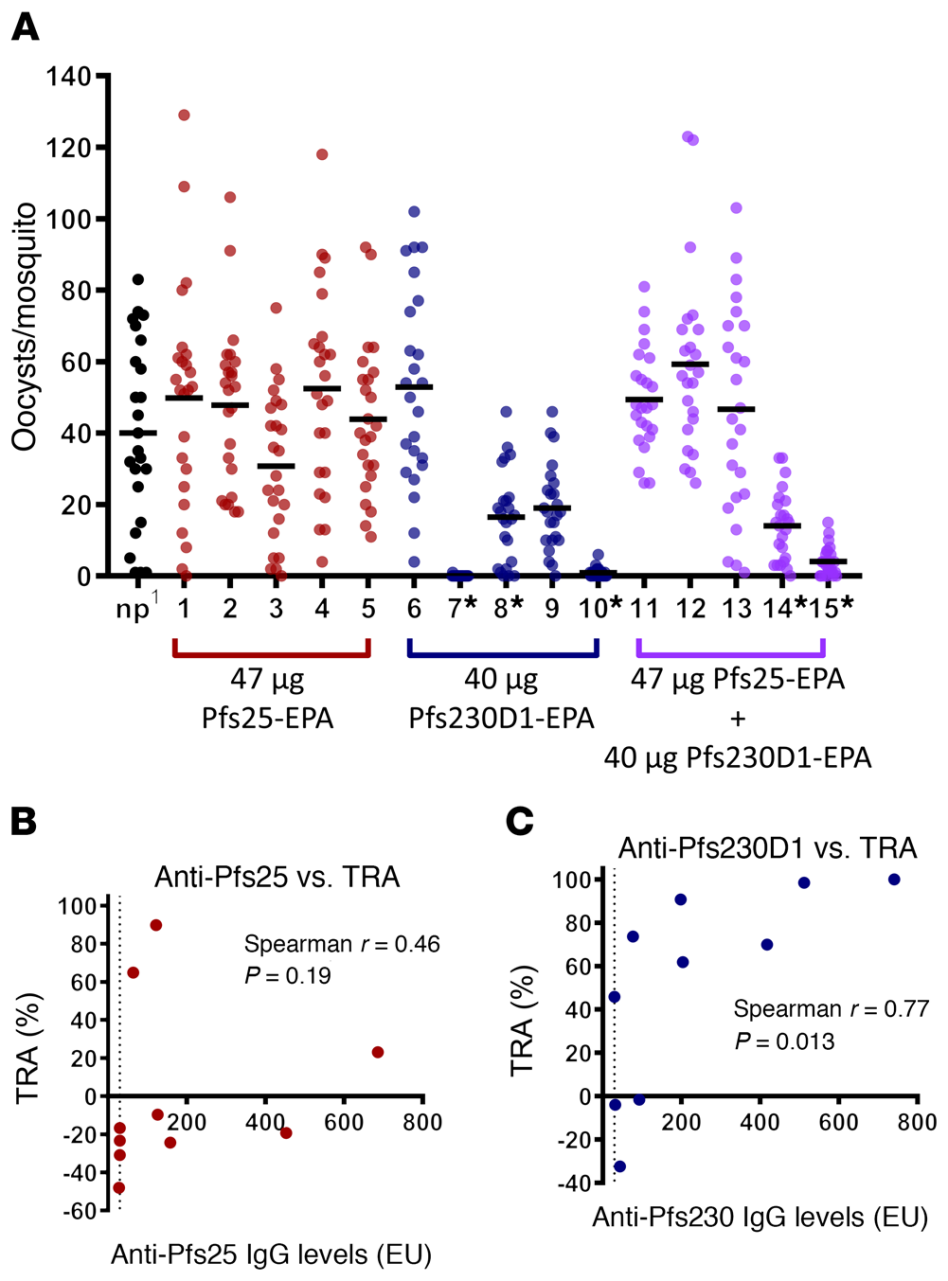
Anti-Pfs230D1 has functional activity after 2 doses in humans. Vaccination induces functional antibodies after 2 doses. Vaccine dosing refers to the amount of target antigen (47 μg Pfs25 or 40 μg Pfs230D1) contained in the total conjugate vaccine dose. Sera from subjects (*n =* 5/group) were collected 2 weeks after the second dose and used for SMFA. Each data point represents the oocyst count from 1 mosquito. np, naive sera pool. *P < 0.05 difference from naive pool; 15 pairwise Kruskal-Wallis tests with Dunn-Bonferroni correction for multiple comparisons. Figure data are also represented in Supplemental Table 5.

Sera from peak antibody levels 2 weeks after the second dose in the high-dose groups were measured for functional activity (TRA and TBA) by SMFA. In general, TRA is used to compare results from different studies owing to consistency between assays, whereas TBA varies between assays based on parasite infectivity to mosquitoes (27). Consistent with previously published data (17) and with preclinical studies, 2 doses of Pfs25-EPA did not induce sufficient antibody to reduce transmission (Figure 3A and Supplemental Table 5). However, aligning with the results seen in rhesus, Pfs230D1 induced high functional activity in humans. TRA was greater than 90% in 2 of 5 individuals from the Pfs230D1-EPA arm (99%, 98%) and greater than 50% in 2 others (73%, 62%), versus the Pfs25 group in which 0 of 5 individuals had TRA greater than 50%, representing a significant improvement of Pfs230D1-EPA over the benchmark Pfs25-EPA candidate (Fisher’s exact test, *P <* 0.05). In the Pfs25 + Pfs230D1 combination group, one individual had 90% and one had 65% TRA. TRA correlated well with anti-Pfs230D1 (*r* = 0.77, *P =* 0.013) but not anti-Pfs25 responses (Figure 3, B and C), demonstrating that the functional activity is due to the Pfs230D1 vaccine. Antibody levels achieved after 2 doses of Pfs25 vaccine were similar in 2 previous studies in malaria-naive US individuals (geometric mean [GM] EU = 147; ref. 17) and malaria-exposed individuals in Mali (GM EU = 93.1; ref. 18) as in this study (GM EU = 160.1), when comparing individuals who received the same vaccine dosage. Functional Pfs25 activity was also similar between the prior US study (17) and this study, with few (2/17) or no (0/5) individuals with serum TRA greater than 50%, respectively, after 2 vaccine doses. Serum TRA was not measured after vaccine dose 2 in the Mali field trial (18) and so cannot be compared with the current study.

**Figure 4:**
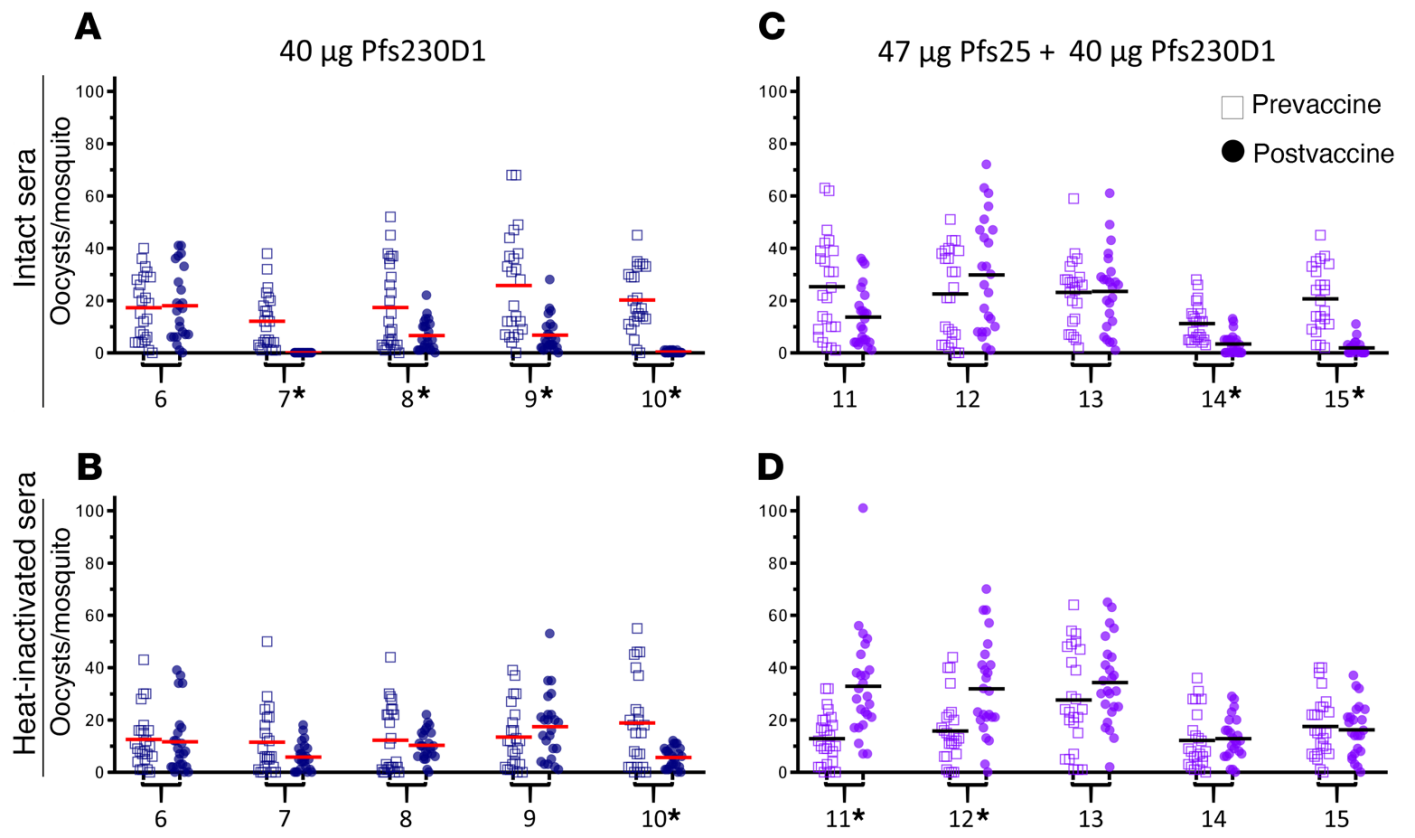
Functional activity of anti-Pfs230 requires active complement in humans. Vaccine dosing refers to the amount of target antigen (47 μg Pfs25 or 40 μg Pfs230D1) contained in the total conjugate vaccine dose. Sera from subjects vaccinated with Pfs230D1-EPA/Alhydrogel or Pfs230D1-EPA+Pfs25-EPA/Alhydrogel were divided in 2 aliquots, and after complement inactivation of 1 aliquot, used for SMFA. Each data point represents the oocyst count from 1 mosquito. *P < 0.05 difference between postvaccine and prebleed sera from individuals, Wilcoxon matched pairs signed-rank test with Bonferroni adjustment. Each panel shows 5 pairwise tests. Figure data are also represented in Supplemental Table 6.

Again, as seen in the nonhuman primate model, dependency on complement was confirmed with the Pf230D1 immune sera samples, as heat-inactivation of serum complement significantly reduced (*P =* 0.009, Wilcoxon matched-pairs signed rank test), but did not completely eliminate, functional activity (Figure 4, A–D, and Supplemental Table 6). Sera from 2 individuals who received the vaccine combination appeared to increase parasite transmission to mosquitoes after heat inactivation (Figure 4D). Enhancement of *P*. *vivax* transmission has also been observed with naturally acquired antibodies or monoclonal antibodies used at low doses in membrane feeding assays (26). Naturally acquired serum transmission-enhancing activity has been observed against both *P*. *vivax* and *P*. *falciparum* (28–30), and this enhancing activity has been most apparent in heat-inactivated sera (29). While this enhancing activity has not previously been associated to Pfs230 antibodies in the absence of complement, our results echo these earlier findings.

We examined antibody isotypes and subclasses to assess the role of complement-fixing antibody. First, we measured Pfs230D1-specific antibody isotypes in sera from rhesus macaques that received either Pfs230D1-EPA/Alhydrogel alone or Pfs25-EPA/Alhydrogel + Pfs230D1-EPA/Alhydrogel combination 2 weeks after the second and third vaccinations, and observed a substantial IgG1 and minimal IgM response (Supplemental Figure 5). Then we measured antibody isotypes and subclasses from human vaccinees 2 weeks after the second vaccination, and as seen in rhesus, the dominant antibody response was IgG1 (Supplemental Figure 6). Two human vaccinees produced appreciable amounts of IgG3, which is also complement-fixing. Pfs230-specific IgM, which also has complement-fixing properties, was readily detected in 4 of the 5 vaccinees but they were consistently lower than the IgG1 isotype in all samples. We surveyed day 84 sera for 5 subjects that had TRA on day 56, and significant functional activity persisted for 4 of 5 subjects (Supplemental Figure 7A). We compared IgG and IgM purified from day 84 sera of 2 subjects (subjects 7 and 10) in SMFA, and oocyst counts were significantly lower in the presence of IgG than IgM (for subject 7, *P =* 0.042; for subject 10, *P =* 0.006; for both subjects combined, *P =* 0.001, Supplemental Figure 7B). Both serum and IgG from subject 7 were tested for membrane attack complex (MAC) formation on gametes, showing MAC formation in assays using intact but not heat-inactivated sera (Supplemental Figure 7, C–F).

## Discussion

A TBV could play a pivotal and unique role in malaria elimination efforts by inducing durable immune responses that interrupt human-to-mosquito malaria transmission over an extended period of time. In our earlier clinical trials, the leading TBV candidate Pfs25 induced functional activity at peak antibody levels, but the activity was lost rapidly as antibodies decreased (17, 18). Here, we sought to increase TBV activity by combining the postfertilization antigen Pfs25 with the prefertilization antigen Pfs230D1. We found that Pfs230D1 was significantly more potent than Pfs25 as a vaccine, and the addition of Pfs25 to Pfs230D1 did not appear to improve on this activity. The superior activity of Pfs230D1 over Pfs25 was predicted by preclinical studies in rhesus but not in mice and was largely explained by complement-dependent activity seen with monkey and human but not rodent antibody. Pfs230 or Pfs230 combination vaccines warrant further evaluation in field studies to assess their potential as a tool for malaria elimination, as do adjuvants that increase levels and durability of functional antibody (31), particularly complement-fixing antibody isotypes.

TBV candidate antigens, including Pfs230, have been challenging to prepare as properly folded proteins, owing to their cysteine-rich sequence and highly folded structure. Importantly, functional antibodies against these antigens commonly target conformation-dependent epitopes (20). The Pfs230 extracellular fragment includes 14 domains with 6-cys structure, a motif that has been particularly difficult to reproduce as recombinant antigen. Earlier studies showed that N-terminal Pfs230 fragments, such as constructs encompassing an upstream region with domains 1 through 3 (called Pfs230-C, ref. 32) or an upstream region with domains 1 and 2 (called Pfs230D1-2, ref. 20), induce functional antibodies in animal studies. Here, we used *Pichia* expression system to generate properly folded Pfs230 domain 1, which induced more potent antibodies in rabbits compared with the Pfs230D1-D2 polypeptide in the ex vivo SMFA (20), after prior success expressing full-length extracellular Pfs25 in *Pichia* (33) that induced functional antibodies in human studies (17). Our preclinical studies consistently demonstrated *Pichia*-expressed Pfs230D1 reacted to conformation-dependent functional monoclonal antibodies (20) and induced functional IgG in immunized animals.

Recent studies in mice suggested that Pfs25 immunogens were similar (13) or superior (14) to Pfs230 immunogens for inducing functional antibodies that block parasite transmission. In mice, we similarly found no significant differences in the ability of Pfs25 and Pfs230D1 recombinant immunogens prepared as conjugate nanoparticle vaccines to induce antibody activity in mice. However, our studies in monkeys suggested that the Pfs230D1 immunogen was significantly more potent for inducing serum functional activity. The difference between models can be explained at least partly by the complement-dependent nature of Pfs230-induced activity, which was pronounced in assays with monkey but not mouse antibody. Early studies using mouse monoclonal antibodies raised against native Pfs230 on gametes revealed functional antibodies restricted to IgG2a and IgG2b subclasses. Complement-mediated lysis was the putative mechanism of transmission blockage (23–25), with the exception of the murine mAb 4F12, isotype IgG1, which has been crystallized in complex with Pfs230D1 (34) and effectively blocks transmission independent of complement (20).

In a previous study, we showed that mice immunized with Pfs230D1 showed a dominant IgG1 isotype response (35), which is noncomplement fixing in mice. Here, we observed in both rhesus and humans that IgG1, which has the highest complement-fixing ability, was the dominant antibody induced. IgG2, IgG3, IgG4, and IgM levels were measured in humans and were consistently lower. Importantly, TRA occurred in subjects with IgG1 dominant responses, and activity was higher in the IgG than the IgM fraction of sera. In addition, both sera and purified IgG induced formation of membrane attack complex (MAC) proteins, indicating activation of the complement cascade. These results are consistent with recent evidence that human monoclonal antibodies we generated from Pfs230D1 vaccinees also induce MAC formation on gametes in the presence of intact but not heat-inactivated sera (36). Others have shown that anti-Pfs230 antibodies increase binding of C1q on sexual-stage parasites, which implicates activation of the classical pathway (37) as the initiating event that ultimately results in MAC formation.

Of note, mouse polyclonal sera induced by vaccination with an alum-based adjuvant conferred activity in the absence of the complement pathway. Additionally, rhesus immune sera significantly reduced transmission in the absence of complement, demonstrating other complement-independent mechanisms, such as neutralization. Indeed, in the clinical trial, function in one subject was not completely ablated in the absence of complement (Figure 4B). We are currently exploring mechanisms by which rodent mAb 4F12 might neutralize gametes in the absence of complement activity.

Our initial goal for the human studies was to examine whether a combination of Pfs25 with the prefertilization antigen Pfs230D1 was safe and might induce more potent and longer-lived activity than Pfs25 alone. In previous rodent studies, a combination of yeast-expressed Pfs25 and Pfs28 induced greater activity than either antigen alone (19), but combinations of Pfs25 and Pfs28 or Pfs25 and Pfs230-C, delivered as virus-vectored vaccines, did not yield additional activity (14). Ultimately, each antigen combination must be tested empirically for additive or synergistic activities in humans. Here, we observed that Pfs230D1 alone formulated in Alhydrogel (an adjuvant with relatively modest immunopotentiating activity) could induce functional serum activity after only 2 doses in some individuals, suggesting vaccine activity could be enhanced by increasing the number of doses or by alternative adjuvants. Further, the addition of Pfs25 to Pfs230D1 during immunization did not provide additional activity, although we saw evidence for both positive and negative effects on Pfs25 responses at different dosages of the vaccine combination. We are currently examining these vaccine antigens and the combinations using alternative adjuvants in field studies in endemic areas (ClinicalTrials.gov ID NCT02334462, NCT02942277, NCT03917654), where malaria exposure can also modify vaccine responses.

Taken together, the data show 2 conclusions: Pfs230D1 is a superior transmission-blocking antigen to Pfs25, and the rhesus model is more predictive of the functional human immune response to Pfs230D1 than is the mouse model. The results from these studies yield valuable information for future studies to understand immune responses to Pfs230D1 and how improvements in vaccine development may lead to a licensed TBV.

## Methods

### Animal studies

Five- to 8-week-old naive, female BALB/c or CD-1 mice were purchased from Taconic Laboratories and maintained at an NIH facility. Immunizations were performed by either intraperitoneal or intramuscular injection in the anterior tibialis in a volume of 50 μL using a standard day 0 and day 28 regimen. *Macaca mulatta* (rhesus) were randomized by age, sex, and weight, and were maintained in an AAALAC-accredited NIAID facility. Vaccinations were performed using doses intended for humans on days 0, 56, and 168 by intramuscular injection in a volume of 0.6 mL (Pfs25-EPA/Alhydrogel) or 0.8 mL Pfs230D1-EPA/Alhydrogel) in the leg, alternating legs for boosting injections. Preclinical mouse formulations were prepared with doses as stated, 50 μL final immunization volume, using a final Alhydrogel content of 1.6 mg/mL in PBS. The nonhuman primate study used clinical doses and/or clinical formulations, with doses and volumes as stated, on 1.6 mg/mL Alhydrogel in PBS. All preclinical formulations were analyzed for antigen binding to Alhydrogel, and all products were 100% bound.

### Human studies

#### Study product.

The PpPfs25M and EcEPA lots, both manufactured at Walter Reed Bioproduction facility in cGMP compliance, were used to manufacture the conjugate. PpPfs25M is a *Pichia*-expressed recombinant Pfs25 with a molecular mass of 18,713 Da. EcEPA is an *E*. *coli*–expressed recombinant protein with molecular mass of 66,975 Da. The Pfs25M-EPA conjugate was produced by reaction between thiolated PpPfs25M and maleimide-activated EcEPA, followed by purification using size-exclusion chromatography. The Pfs25M-EPA conjugate was manufactured at Walter Reed Bioproduction facility in cGMP compliance in August 2013. Pfs25M-EPA/Alhydrogel was manufactured at Walter Reed Bioproduction facility in cGMP compliance in July 2014. Each single-use vial contained 78 μg/mL conjugated Pfs25M, 78 μg/mL conjugated EPA, and 1600 μg/mL Alhydrogel in a volume of 0.8 mL. The vial label read: 78 μg/mL conjugated Pfs25M on Alhydrogel.

The Pfs25M-EPA/Alhydrogel vaccine was provided as a single-use vial. A 0.2-mL volume was administered for delivery of 16 μg conjugated Pfs25M, 16 μg conjugated EPA, and 320 μg Alhydrogel. A 0.6 mL volume was administered for delivery of 47 μg conjugated Pfs25M, 47 μg conjugated EPA, and 960 μg Alhydrogel. The vaccine was drawn into the syringe up to 5 hours prior to administration and mixed by hand before injection to ensure resuspension.

The PpPfs230D1M and EcEPA lots, both manufactured at Walter Reed Bioproduction facility in cGMP compliance, were used to manufacture the conjugate. PpPfs230D1M is a *Pichia-*expressed recombinant subsegment (S_542_–G_736_) of Pfs230 with a molecular mass of 21,854 Da. EcEPA is an *E*. *coli*–expressed recombinant protein with molecular mass of 66,975 Da. The Pfs230D1M-EPA conjugate was produced by reaction between thiolated PpPfs230D1M and maleimide-activated EcEPA, followed by purification using size-exclusion chromatography. The Pfs230D1M-EPA conjugate was manufactured at Walter Reed Bioproduction facility in cGMP compliance in August 2013.

The Pfs230D1M-EPA/Alhydrogel vaccine was formulated in cGMP compliance in July 2014 and provided as a single-use vial. A 0.1 mL volume was administered for delivery of 5 μg conjugated Pfs230D1M, 5 μg conjugated EPA, and 160 μg Alhydrogel. A 0.3 mL volume was administered for delivery of 15 μg conjugated Pfs230D1M, 15 μg conjugated EPA, and 480 μg Alhydrogel. A 0.8 mL volume was administered for delivery of 40 μg conjugated Pfs230D1M, 40 μg conjugated EPA, and 1280 μg Alhydrogel. The vaccine was drawn into the syringe up to 5 hours prior to administration and mixed by hand before injection to ensure resuspension.

Pfs230D1M-EPA/Alhydrogel was manufactured at Walter Reed Bioproduction facility in cGMP compliance in July 2014. Each single-use vial contained 50 μg/mL conjugated Pfs230D1M, 49 μg/mL conjugated EPA, and 1600 μg/mL Alhydrogel in a volume of 1.0 mL. The vial label read: 50 μg/mL conjugated Pfs230D1M on Alhydrogel. Alhydrogel is an aluminum hydroxide gel and has been extensively used as an adjuvant in licensed human vaccines. Alhydrogel was supplied as a sterile product in water without preservatives.

For both vaccines, the total vaccine dose in humans was intended not to exceed 100 μg of the vaccine conjugate, and therefore the amount of target antigen was based on the mass ratio of target antigen to carrier protein, resulting in 47 μg for Pfs25 for the Pfs25-EPA conjugate, and 40 μg Pfs230 for the Pfs230-EPA conjugate. Pfs25M-EPA/Alhydrogel and Pfs230D1M-EPA/Alhydrogel were stored at 2°C to 8°C. Vials were transported and stored in temperature-controlled conditions, as per standard operating procedures. Temperature data loggers accompanied the vaccines at all times to ensure storage temperature limits had not been violated. Vaccines and adjuvant were not frozen at any time, and refrigerator temperature was continuously monitored.

#### Clinical study procedures.

The clinical study was designed as an open-label dose-escalation study to examine the safety and immunogenicity of Pfs230D1-EPA/Alhydrogel and Pfs25M-EPA/Alhydrogel alone or coadministered. The initial open-label dose-escalating, 2-dose regimen (0, 1 month; *n =* 5/group) was performed in the US prior to a larger double-blinded study conducted in Mali. Participants were sequentially enrolled. No blinding or placebo arms were implemented. No randomization occurred.

Vaccines were administered as intramuscular injections into the deltoid muscle. Arms were alternated with successive vaccinations if a single vaccination was given. If simultaneous vaccinations were administered (2 individual vaccinations at the same time), each vaccine was drawn up and delivered separately, in alternate arms. When choosing an arm for the vaccine injection, clinicians considered whether there was an arm injury, local skin problems such as scarring or rash, or significant tattoo that precluded administering the injection or would interfere with evaluating the arm after injection. In keeping with the NIH Clinical Center policy, 11. Malaria Research and Training Center practices and procedures, and good medical practice, acute medical care was provided to subjects for any immediate allergic reactions or other injury resulting from participation in this research study.

#### ELISA.

Immulon 4 HBX flat bottom microtiter plates (Dynex Technologies) ELISA plates were coated with 1 μg/mL of antigen in a volume of 100 μL per well in carbonate coating buffer (pH 9.6) overnight at 4°C. After blocking in 5% skim milk in TBS blocking buffer in a volume of 320 μL per well for 2 hours, samples were serially diluted in TBS/5% milk and plated in triplicate in a volume of 100 μL per well and incubated at room temperature for 2 hours. Plates were washed 4 times and alkaline phosphatase–labeled goat anti-mouse IgG (H+L), goat anti-human IgG (H+L), or goat anti-monkey phosphatase labeled secondary antibody (Seracare Life Sciences, catalog 5220-0303) was added in a volume of 100 μL per well and incubated at room temperature for 2 hours. After washing 4 times, dissolved phosphatase substrate tablets (MilliporeSigma) were added in a volume of 100 μL per well and plates were incubated for 20 minutes before the optical density (OD) was measured with a Spectramax 340PC (Molecular Devices). Each ELISA plate contained an internal serum standard from which a 4-parameter curve was calculated with Softmax software. According to laboratory standard operating procedures, any samples for which ELISA results from triplicate wells exceeded a prespecified coefficient of variation were repeated. ELISA units were assigned to test samples based on the sera dilution that gave an OD of 1.0, adjusted to the internal standard. For the Pfs230 isotyping assays, similar procedures were followed and the list of detecting antibodies used are listed in Supplemental Table 7.

#### Transmission-blocking and -reducing activity.

TBA (reduction in infection prevalence) and TRA (reduction in infection intensity) of the sera were tested by an ex vivo SMFA as previously described (13). Briefly, an in vitro 14- to 16-day-old gametocyte culture of *P*. *falciparum* (NF54 line) was evaluated for stage V gametocytes (> 0.5%) and the presence of exflagellation centers observed at ×400 magnification (> 1 per field). The culture was diluted with washed O^+^ RBCs from a malaria naive donor (Interstate Blood Bank, Memphis, Tennessee, USA) to achieve 0.12% ± 0.05% concentration of stage V gametocytes. For each sample, 100 μL of the pelleted diluted culture (100% hematocrit) was mixed with 160 μL test serum. For human sera samples, 160 μL was used neat. For rhesus sera samples, 60 μL of test sera was mixed with 100 μL of a pool of naive human AB^+^ sera. For mouse sera samples, 20 or 30 μL of test sera was mixed with 130 μL of pooled naive sera. All samples were immediately fed to prestarved (~24 hours) 3- to 8-day-old *Anopheles stephensi* (Nijmegen strain) mosquitoes through a Parafilm membrane stretched across a glass mosquito feeder kept warm by a circulating water membrane at 40°C. Test sera were not heat inactivated. After feeding, mosquitoes were maintained for 8 days at 27°C and 80% humidity conditions to allow for the development of parasites. Infectivity was measured by dissecting at least 20 gravid mosquitoes per sample, staining the midguts with 0.05% mercurochrome solution in water for 20 minutes and counting the number of oocysts on each midgut. The feeding experiment was not analyzed unless the average oocyst count in the assay control mosquitoes (at least 20 dissected mosquitoes fed with naive heat-inactivated serum) was more than 4. The TBA and TRA were calculated by the following formulas:


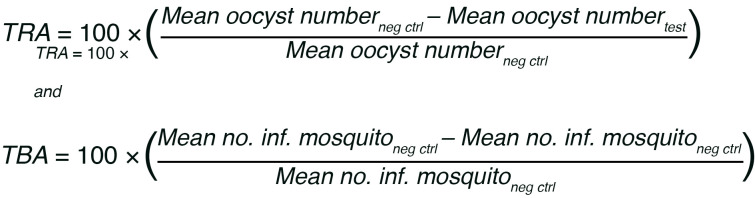


Equation 1

where the negative control (neg ctrl) feed used pooled prevaccination sera from all subjects. Each sample from monkeys or humans who received Pfs230 or Pfs230 + Pfs25 vaccine was tested in 2 independent feeding experiments and these 2 TRA values were averaged to obtain a single subject level TRA for a given time point.

#### Immunofluorescence assay to assess the deposition of the MAC.

Formation of MAC and further activation of the complement system were assessed using immunofluorescence of live female gamete parasites. Briefly, 5 mL of a *P. falciparum* NF4 gametocyte stage V culture were centrifuged at 500*g* for 5 minutes and added to an exflagellation medium containing 900 μL RPMI, 100 μL PBS, and 1 μL xanthurenic acid, then left for 1.5 hours at room temperature. Cells were resuspended in 5 mL RPMI and applied to a 15 mL Nycodenz gradient (16%, 11%, and 6%), then centrifuged at 7000*g* for 30 minutes. Parasites located in the interface between 6% and 11% were collected into 50 mL RPMI and spun down at 2000*g* for 10 minutes. Parasites were then incubated with serum or 100 μg/mL total IgG previously purified from subject 30G and diluted in PBS. IgG1 isotype control with heavy and kappa chains was purchased from Creative Biolabs. The suspension was incubated at 37°C for 45 minutes with either 50 μL of intact sera or sera heat-inactivated at 56°C, both obtained from a healthy US donor. Subject serum was used directly without supplementation. During the incubation, the tubes were gently mixed every 10 minutes to facilitate C5b-9 and C5b-8 deposition on cells. A quantity of 2 mL cold PBS was used to stop the reaction and to wash the cells. Suspension was centrifuged at 500*g* for 5 minutes and the pellet was then incubated with 10 μg/mL of the mAb anti-C5b-9 + C5b-8 (Abcam, catalog ab66768) for 2 hours on ice. Cells were washed with PBS, centrifuged at 500*g* for 5 minutes, and stained with Hoechst 33342 Solution (Thermo Fisher Scientific) diluted at 1:20,000 for 8 minutes and further washed with PBS. Cells were kept in parasite culture media until the imaging was performed in a TCS SP8 MP microscope (Leica) at 37°C. Quantification analyses were performed assessing the mean fluorescence per nuclei stained.

#### Statistics.

All statistical tests were performed using Prism v7.0 by GraphPad Software. Statistical analyses were conducted using the following tests: Pearson’s correlation coefficient, Wilcoxon rank sum tests with continuity correction, Kruskal-Wallis rank sum tests with and without Dunn-Bonferroni adjustment, Spearman’s coefficient, and Wilcoxon matched pairs signed-rank tests. Figure legends denote exact tests used for individual panels. *P* values less than 0.05 were considered significant; *P* values were corrected for multiple comparisons where appropriate.

#### Study approval.

All animal procedures were performed according to protocols approved by the NIAID and NIH Animal Care and Use Committee. All procedures were in accordance with the *Guide for the Care and Use of Laboratory Animals* (National Academies Press, 2011). This open-label phase 1 trial was performed at the NIH Clinical Trial Center in Bethesda, Maryland, USA. The study was conducted under an investigational new drug (IND) application with the US Food and Drug Administration (#16251). The protocol was approved by the IRB of the NIAID and is registered at ClinicalTrials.gov under trial investigation number NCT02334462. All participants gave written informed consent in order to participate in the study.

## Author contributions

SAH, CA, EEG, KMR, YW, and PED designed and conceptualized the study. KMR, DZ, RH, PVS, NJM, LEL, CHC, JPR, YW, and DLN conducted the experiments. SAH, CA, AM, HD, CVH, OM, and IZ conducted clinical investigation. CA, BJS, and IZ analyzed the results. CA, BJS, and EEG curated the data. SAH and CA visualized data and wrote the first manuscript draft. All authors reviewed and edited the manuscript. SAH and PED supervised the clinical trial. PED supervised all teams.

## Supplementary Material

Supplemental data

Trial reporting checklists

ICMJE disclosure forms

## Version 1 02/09/2021

In-Press Preview

## Version 2 04/01/2021

Electronic publication
